# Skin Sparing Mastectomy with Preservation of Nipple Areola Complex and Immediate Breast Reconstruction in Patients with Breast Cancer: A Single Centre Prospective Study

**DOI:** 10.1155/2014/589068

**Published:** 2014-11-11

**Authors:** Debarati Chattopadhyay, Souradip Gupta, Prabir Kumar Jash, Marang Buru Murmu, Sandipan Gupta

**Affiliations:** ^1^Department of Plastic Surgery, IPGME&R, Kolkata, India; ^2^Department of Plastic Surgery, Medical College Kolkata, 88 College Street, Kolkata, West Bengal 700073, India; ^3^Department of Surgery, Midnapore Medical College, India

## Abstract

*Background.* Skin and nipple areola sparing mastectomy (NASM) has recently gained popularity as the management of breast cancer. This study aims to evaluate the aesthetic outcome, patient satisfaction, and oncological safety of NASM. * Methods.* The study prospectively analyzes the results of NASM and immediate breast reconstruction in 34 women with breast cancer. The criteria for inclusion were core biopsy-proven, peripherally located breast cancer of any tumor size and with any “N” status, with documented negative intraoperative frozen section biopsy of retroareolar tissue, and distance from the nipple to tumor margin >2 cm on mammography. *Results.* The median age of the patients was 45 years. The majority had either stage II or stage III breast cancer. The median mammographic distance of tumor from nipple areola complex (NAC) was 3.8 cm. The overall operative morbidity was minimal. The NAC could be preserved in all the patients. There was no local recurrence of tumor at median follow-up of 28.5 months. The aesthetic outcomes were satisfactory. *Conclusion.* NASM and immediate breast reconstruction can be successfully achieved with minimal morbidity and very low risk of local recurrence in appropriately selected breast cancer patients, with acceptable aesthetic results and good patient satisfaction.

## 1. Introduction

Modified radical mastectomy is a disfiguring operation that is associated with considerable psychological trauma for the affected woman. A woman diagnosed with breast cancer fears not only for her life but also the mutilation of her body. On the other hand women undergoing breast conserving surgery (BCS) live with constant anxiety of harbouring the residual malignancy within. Research to find a feasible alternative has led to the renewal of interest in skin sparing and nipple areola complex sparing mastectomy (NASM) which entails the removal of the breast tissue while preserving the natural skin envelope as much as possible.

First described by Freeman in the 1960s, NASM was traditionally utilized for benign breast lesions [1]. Although there were sporadic reports of mastectomy with NAC preservation for breast cancer treatment in the 1980s the technique fell into disuse during subsequent years due to controversies about its oncologic safety [1–3].

The resurgence of this procedure as the primary management in breast cancer has been made possible by a number of studies which have concluded that the nipple areola complex involvement by breast cancer has been overestimated in the past. The results of studies worldwide suggest that preservation of the nipple areola complex with immediate breast reconstruction is oncologically safe in carefully selected patients, with superior aesthetic outcome. However, review of the literature has revealed scarcity of data from India on this subject.

The present study prospectively analyzed the results of skin sparing mastectomy with nipple areola complex preservation and immediate breast reconstruction in women with breast cancer in a teaching hospital in India. A total of 34 patients were studied with assessment of the aesthetic outcomes, patient satisfaction with the results of surgery, and evaluation of the oncological safety of the surgical procedure in terms of local recurrence of breast cancer.

### 1.1. Aims and Objectives of the Study

1. To evaluate the aesthetic outcome in women undergoing nipple areola and skin sparing mastectomy (NASM) for breast cancer and immediate breast reconstruction.
2. To assess patient satisfaction after the surgical procedure.
3. To evaluate the oncological safety of the surgical procedure in terms of local recurrence of breast cancer.

## 2. Materials and Methods

The present study prospectively analyzed the results of NASM and immediate breast reconstruction in 34 patients with breast cancer attending the Department of Plastic Surgery, Medical College Kolkata, India, over a period of twenty-one months between April 2011 and December 2012. The criteria for inclusion were core biopsy-proven, peripherally located breast cancer of any tumour size and with any “N” status, located >2 cm away from margin of areola, with documented negative intraoperative frozen section biopsy of retroareolar tissue, and distance from the nipple to tumour margin >2 cm on mammography or high-resolution ultrasonography. Patients having a central quadrant tumour, a tumour encroaching within 2 cm of areolar margin, a tumour fixed to the chest wall, clinical suspicion of nipple areola involvement, or inflammatory breast cancer were excluded from the study.

The parameters to be studied were prefixed as follows:

1. aesthetic outcome: this was stratified by subscales according to Lowery et al. [4]. Volume, contour, placement of breast mound, and inframammary fold were evaluated with zero to 2 points for each parameter. Results were defined as excellent: 7 to 8 points, good: 6 to 6.9 points, fair: 5 to 5.9 points, and poor: <5 points;
2. patient satisfaction: the scoring was done by the patient along a scale of 1 to 10 where score 1 to 4 was categorized as poor, 5 to 6 as fair, 7 to 8 as good, and 9 to 10 as excellent;
3. oncological safety: local recurrence was defined as histologically proven recurrent tumor occurring in either the ipsilateral breast skin or the nipple areola complex.

### 2.1. Study Tools and Technique

#### 2.1.1. Operative Technique

For nipple areola sparing and skin sparing mastectomy (NASM), either lateral incision or inframammary incision was used. In case of inframammary incision the axillary nodal dissection was done through a separate vertical or inverted hockey stick like incision in the axilla. After skin incision, the breast tissue was dissected from the pectoralis fascia by sharp dissection. The dissection was then carried in the subdermal plane. The skin flap thickness varied from 2 to 5 mm and consisted of 1-2 mm of intact dermis and a thin layer of subcutaneous fat. The base of the nipple was divided sharply. The nipple papilla was not cored out.

#### 2.1.2. Frozen Section Biopsy

Intraoperative frozen section biopsy was taken from two sites. A total of 5 samples were taken in each case:

- two samples from the glandular tissue under the areola,
- one sample from the nipple base,
- two samples from the subcutaneous tissue overlying the tumour.

All the frozen section biopsy samples were interpreted by the same pathologist in the Department of Pathology, Medical College Kolkata. The NAC was preserved only when palpation, shape, and color of the nipple were normal and when intraoperative frozen section biopsy from under the NAC was tumor-free. If the subcutaneous tissue overlying the tumor was found to be positive on frozen section biopsy an incision was placed over the tumor site and a skin island was dissected with the breast specimen to achieve distant tumor-free margins.

#### 2.1.3. Breast Reconstruction

After the completion of the mastectomy and appropriate axillary clearance, all patients underwent immediate breast reconstruction with either (i) autologous tissue: the transverse rectus abdominis myocutaneous (TRAM) flap or the latissimus dorsi myocutaneous flap, or (ii) by the placement of a permanent silicone gel implant.

#### 2.1.4. Follow-Up

Adjuvant systemic treatment was administered according to the NCCN guidelines. The final aesthetic results were evaluated at 6 months postoperatively and stratified by subscales according to Lowery et al. [4]. The patients were followed up regularly for at least 18 months after surgery.

## 3. Results

The median age of the patients was 45 years (range: 28–61 years). Majority of patients (55.9%) were in the age group of 41 to 50 years. At the time of diagnosis, 13 patients had TNM stage IIIA breast cancer, 19 patients had stage II cancer, and only one patient had stage I cancer. All the patients had invasive ductal carcinoma, except a single patient who had ductal carcinoma in situ (DCIS) (Table 1). The distance of tumour from the nipple areola complex as seen on mammography was between 2 cm and 4 cm in about two-thirds of the patients in our study, and the median distance of the tumour from NAC was 3.8 cm (range: 2.4–5.2 cm). Intraoperative frozen section studies of retroareolar tissue and the subcutaneous tissue immediately above the tumour were performed to decide whether the NAC should be preserved or not. No involvement of the nipple core or areola was found on frozen section biopsy in any patient. Frozen section biopsy was positive from the subcutaneous tissue immediately above the tumour in 5 cases (14.7%). The mastectomy incisions used were mainly inframammary (67%) and lateral (17.6%) incisions. Immediate breast reconstruction using autologous tissue was performed in more than 90% of cases (TRAM flap in 55% and latissimus dorsi myocutaneous flap in 36% cases), whereas silicone implants were used in 8.8% of cases only. The patients received adjuvant systemic treatment as per standard practice guidelines. The overall operative morbidity was minimal with only a few minor complications. The nipple areola complex could be preserved in all the cases (Table 2).

**Figure 1:**
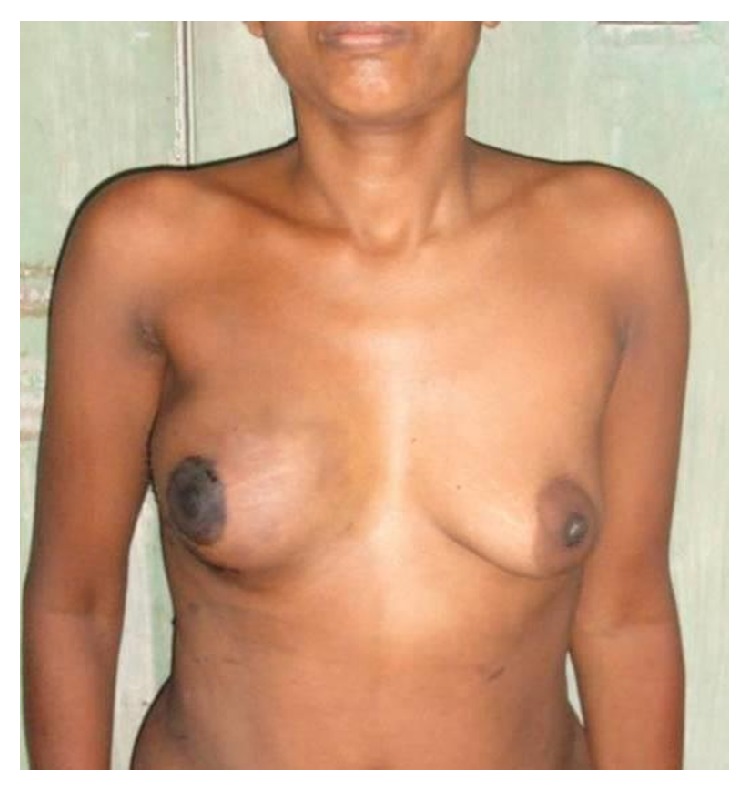
Follow-up of patient after NASM and TRAM flap reconstruction of right breast.

**Figure 2:**
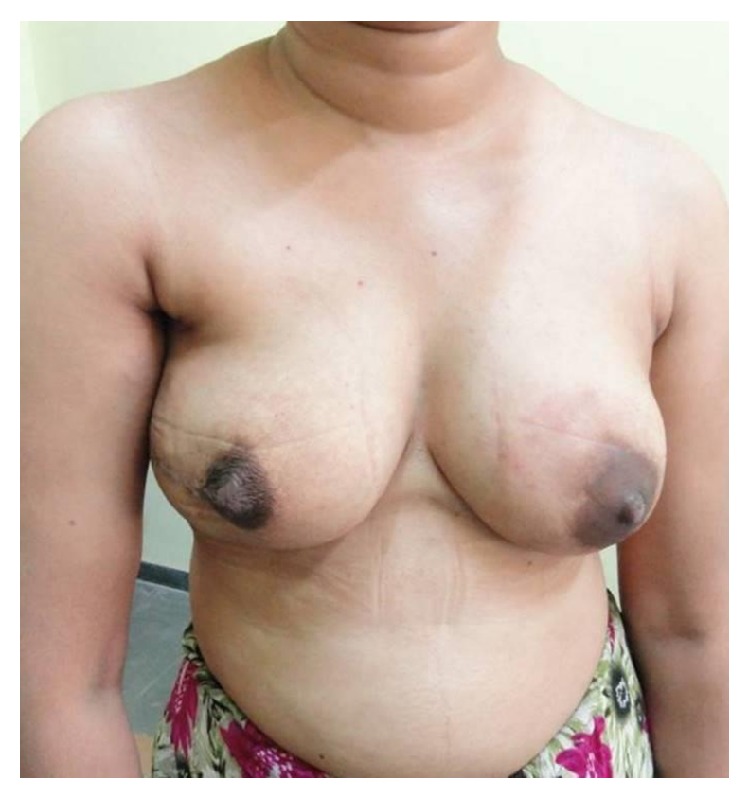
Follow-up of patient after NASM and implant reconstruction of right breast.

The median follow-up was 28.5 months (range: 18−38 months). There was no local recurrence of tumour at follow-up. The aesthetic outcome was excellent in 50% of cases and good in 41.2% of cases (Table 3). Patient satisfaction with results of surgery was excellent in 35.3% of cases and good in 50% of cases. Figures 1 and 2 depict the aesthetic outcome of nipple areola and skin sparing mastectomy after 6 months.

## 4. Discussion

The technique of NASM involves a combination of a skin sparing mastectomy with preservation of the NAC [5]. There have been various attempts over the years to define the selection criteria for NSM [1, 6–12]. There is still no consensus on the indications for this procedure [1]. Recent multivariate models for patient selection for NASM have reported tumour size, stage, and tumour distance from the nipple as some factors predictive of occult nipple involvement [13, 14]. We followed the criteria used by Garcia-Etienne et al. [1]. However, further studies and longer follow-up are necessary to refine the selection criteria for NASM.

Loewen et al. have shown that mammographic distance between the tumor and the nipple is independently predictive of NAC involvement [12]. Some authors have excluded patients from undergoing NASM if imaging (mammography or MRI) showed evidence of tumour within 2 cm of the nipple [15]. The distance of the tumour from the nipple areola complex as seen on mammography was between 2 cm and 4 cm in about two-thirds of the patients, and the median distance of the tumour from NAC was 3.8 cm (range: 2.4–5.2 cm) in our study.

The sensitivity and specificity for frozen section biopsy to detect malignant cells in the retroareolar region has been reported as 90.9% and 98.5%, respectively [16]. In our study, no involvement of the nipple core or areola was found on frozen section biopsy in any patient. Hence, the NAC could be preserved in all the cases. However, frozen section biopsy was positive from the subcutaneous tissue immediately above the tumour in 14.7% cases.

The inframammary incision was used for skin sparing mastectomy in 67.7% of cases in our study. A lateral incision was used in 17.6%, and in those patients who had positive frozen section biopsy of the subcutaneous tissue immediately above their tumour, an additional incision in the skin overlying the tumour was required. Various incisions have been used for NASM by different authors in their reported series [5, 15, 17, 18]. Garwood et al. in their study of total skin sparing mastectomy found that the use of inframammary incisions is an excellent approach for small- or medium-sized breasts and, if enlarged, works well for large breasts as well [19]. Crowe et al. noted that medial incisions may compromise blood flow to the nipple, whereas in all cases performed through a lateral incision, the NAC remained fully intact [18].

Immediate breast reconstruction using autologous tissue and/or silicone implants has been advocated for breast reconstruction after NASM in various studies [20, 21]. In our study, more than 90% of the patients underwent immediate breast reconstruction with autologous tissue (either TRAM or LDMC flap) and implants were used in only few cases. The reason for such a trend could be the financial issues related to the socioeconomic background of our patients. Use of implants for breast reconstruction in our study was preferred mainly in younger patients with nonptotic breasts and in those with early breast cancer where radiotherapy would not be required.

The overall incidence of complications in the study was minimal, and there were few minor complications like seroma, wound infection, and partial umbilical necrosis after TRAM flap in one case each. There was no flap necrosis in any patient. Necrosis of the NAC is a known complication of NASM, with reported rates of 6.7% to 15.8% for any degree of necrosis [22]. In our study only one patient developed partial desquamation of the nipple areola complex, and there was no NAC loss.

All the patients in our study were followed up for at least 18 months after surgery, and the median follow-up period was 38.5 months (range: 18–38 months). There was no local recurrence of tumour in the present study at a median follow-up of 28.5 months. This low rate of local recurrence is supported by the results of previous studies which have confirmed the oncological safety of NASM. For example, in the single centre study of NASM in 95 patients with early breast cancer reported by Omranipour et al. (2008), local recurrence was seen only in one patient (1.1%) and systemic recurrence was seen in two patients (2.1%) at a median follow-up of 69 months, and the authors concluded that NSM is oncologically safe for early breast cancer (stages 0–II) [23]. Sookhan et al. successfully preserved the NAC in 18 cases with no local recurrence at a median follow-up of 10.8 months [5]. Caruso et al. reported only 2% local recurrence rate within the NAC in a series of fifty NASMs for breast cancer after a mean follow-up of 5.5 years [24]. Garcia-Etienne et al. reviewed 1826 procedures of NASM performed for breast cancer treatment published in the recent literature and found only three local recurrences (0.16%) within the NAC [1]. Ubirubu et al. reported three cases of local recurrence at the needle biopsy site in patients treated with SSM whose diagnoses were obtained through stereotactic needle biopsy [25]. Fortunately, there was no evidence of tumour recurrence at the site of needle biopsy in any of our patients. Rusby et al. have published the most recent review of NASM in the literature [14]. They also found recurrence rates of less than 5% in properly selected patients undergoing NASM for breast cancer treatment. Kim et al. (2010) in their retrospective study of 520 patients further widened the indications of NASM [26]. The indications for NASM in their study were any stage, any tumor size, and any tumor areola distance, provided the shape, color, and palpation of the nipple were normal. The locoregional recurrence rates were similar for NASM and mastectomy patients. Salhab et al. found that skin sparing mastectomy and immediate breast reconstruction for operable breast cancer are associated with a high level of patient satisfaction and low morbidity [27]. The procedure seems to be oncologically safe, even in patients with high-risk (T3 or node-positive) carcinoma.

The aesthetic outcomes of skin sparing mastectomy with NAC preservation in our study were evaluated by clinical and photography-based assessments. The aesthetic result was stratified by subscales proposed by Lowery et al. [4]. Volume, contour, placement of breast mound, and inframammary fold were evaluated, and results were defined as excellent, good, fair, and poor according to the total score. Various subjective and objective scores have been used evaluating aesthetic outcomes after immediate breast reconstruction following NASM. Salhab et al. assessed the patient's satisfaction with the outcome of surgery with a detailed questionnaire including a linear visual analogue scale ranging from 0 (not satisfied) to 10 (most satisfied) [27]. Salgarello et al. evaluated the reconstructive and aesthetic outcomes by clinical examinations and by reviewing the clinical pictures of the breasts [28]. More than 90% of patients in our study had good or excellent Lowery scores at 6 months of follow-up. Moreover, the patient satisfaction as assessed by questioning the patients about their satisfaction with the aesthetic results of surgery was acceptable in all the cases.

## 5. Conclusion

We conclude that skin sparing mastectomy with preservation of nipple areola complex and immediate breast reconstruction can be successfully achieved with minimal morbidity and very low risk of local recurrence in appropriately selected breast cancer patients, with acceptable aesthetic results and good patient satisfaction.

## Figures and Tables

**Table 1:** Breast cancer stage at diagnosis.

Stage of breast cancer	Number of cases	Percentage
0 (DCIS)	1	2.9
I	1	2.9
IIA	7	20.6
IIB	12	35.3
IIIA	13	38.3

Total	34	100

**Table 2:** List of complications in postoperative period.

Complication	Number of patients	Percentage
Seroma	2	5.8%
Partial desquamation of NAC	1	2.9%
Wound infection	1	2.9%
Partial umbilical necrosis (after TRAM flap)	1	2.9%

**Table 3:** Results of aesthetic outcome in terms of Lowery scale.

Lowery score	Number of patients	Percentage
Excellent (score 7-8)	17	50%
Good (score 6–6.9)	14	41.2%
Fair (score 5–5.9)	3	8.8%
Poor (score < 5)	0	0

Total	34	100

## Abbreviations

BCS: Breast conserving surgery
NASM: Nipple areola and skin sparing mastectomy
NAC: Nipple areola complex
TRAM: Transverse rectus abdominis myocutaneous.
